# Mining Activities Have Exposed Individuals of an Indigenous Tribe in Brazil to Toxic and Potentially Toxic Elements

**DOI:** 10.1007/s40615-026-02918-y

**Published:** 2026-03-16

**Authors:** Nathália Mariana Santos Sansone, Lucas Silva Mello, Luiz Felipe Azevedo Marques, Fernando Augusto Lima Marson

**Affiliations:** 1 Laboratory of Molecular Biology and Genetics, Postgraduate Program in Health Sciences, Postgraduate Program in Health Data Science, São Francisco University (USF of the Portuguese Universidade São Francisco), Avenida São Francisco de Assis, 218, São Paulo 12916 900 Bragança Paulista, Brazil; 2 Laboratory of Clinical and Molecular Microbiology, Postgraduate Program in Health Sciences, Postgraduate Program in Health Data Science, São Francisco University (USF of the Portuguese Universidade São Francisco), Avenida São Francisco de Assis, 218, São Paulo 12916 900 Bragança Paulista, Brazil; 3 LunGuardian Research Group — Epidemiology of Respiratory and Infectious Diseases, Postgraduate Program in Health Sciences, Postgraduate Program in Health Data Science, São Francisco University (USF of the Portuguese Universidade São Francisco), Avenida São Francisco de Assis, 218, São Paulo 12916 900 Bragança Paulista, Brazil

**Keywords:** Indigenous peoples, Mining, Potentially toxic element exposure, Public health, Vulnerable population

## Abstract

The intensification of mining activities in the Brazilian Amazon has generated profound socio-environmental impacts, particularly on Indigenous populations. This study assessed the levels of contamination by toxic and potentially toxic elements in 720 individuals from the Xikrin do Cateté Tribe, located in the State of Pará, Brazil, a region heavily affected by copper (Cu), gold (Au), and nickel (Ni) mining. The data are part of the public documentation associated with Civil Public Action n° 1,001,462 − 67.2025.4.01.3901. The results showed that 98.5% of the participants had excessive concentrations of at least one toxic or potentially toxic element in their bodies, and 99.7% had at least one chemical element above the recommended limit. The prevalence of excessive chemical elements in the population revealed that manganese (Mn) was the most frequent, detected in 97.0% of individuals, followed by Ni (40.0%), iron (Fe, 32.5%), and cobalt (Co, 19.0%), the latter being a typical geochemical marker of Ni mining. Highly toxic metals with no known physiological function were also detected, including aluminum (Al), barium (Ba), beryllium (Be), lead (Pb), mercury (Hg), titanium (Ti), and Arsenic (As), a semimetal, with prevalence reaching up to 37.0% of the population. All 121 children analyzed showed contamination by multiple toxic and potentially toxic elements, including a one-year-old child with significant exposure to six different metals. In total, 59.1% of the sample exhibited five or more chemical elements at excessive levels. These findings highlight ongoing environmental contamination, potentially associated with regional mining activities. Limited mitigation and remediation efforts may pose risks to the health and well-being of vulnerable populations.

**Graphical Abstract:**

**Figure:**
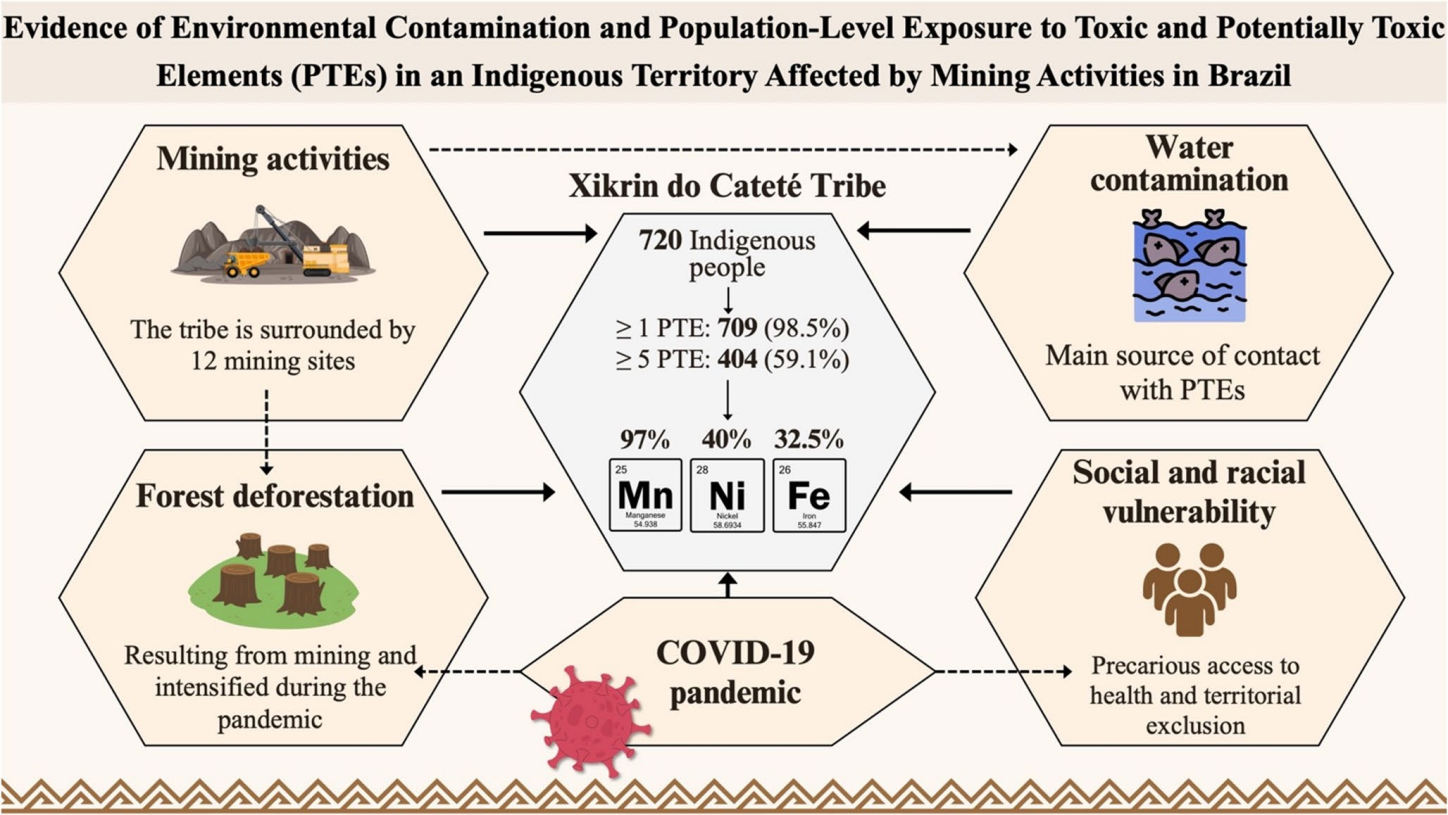
Mining activities in the Brazilian Amazon have resulted in chronic environmental contamination affecting Indigenous peoples. This study analyzed 720 individuals from the Xikrin do Cateté Tribe, located in the State of Pará, Brazil revealing that 98.5% had elevated levels of at least one toxic or potentially toxic element (PTE), with 59.1% exhibiting five or more. The most prevalent PTEs included, for example, manganese (Mn, 97.0%), nickel (Ni, 40.0%), and iron (Fe, 32.5%). Also, 121 children assessed were exposed to multiple PTEs. These findings underscore the urgent need for environmental remediation and public health interventions in mining-impacted territories. %: percentage, COVID-19: coronavirus disease 2019.

**Supplementary Information:**

The online version contains supplementary material available at 10.1007/s40615-026-02918-y.

## Mining, Toxic and Potentially Toxic Elements, and Environmental Contamination in the Amazon

Anthropogenic activities related to environmental exploitation and the ongoing process of industrialization have threatened global sustainability by contaminating water resources, air, and soil with chemical substances at levels exceeding regulatory limits. Among the various industrial activities posing environmental risks, mining stands out as particularly impactful [1]. Although it plays an important role in the industrial development of a country, mining is associated not only with the contamination of aquatic and terrestrial ecosystems but also with direct environmental impacts such as deforestation and hydromorphological alterations in rivers. The primary direct consequence of mining activities is the release into the environment of so-called “heavy metals”, which are characterized by their high atomic weight and density, typically greater than 5–7 g·cm⁻³, depending on the source, and are considered toxic to humans and other living organisms [2, 3]. Mining has been classified as one of the most harmful sources of pollution in the Amazon, a region that host the greatest biodiversity on the planet and plays a key role in global climate regulation [4].

The term “heavy metals” has been used inconsistently over the years. The earliest recorded use in English literature appears in Bjerrum’s *Inorganic Chemistry* (1936), according to the Oxford English Dictionary, where it was defined based on elemental density. Over time, this definition proved inadequate, as density was found to be poorly related to a metal’s chemical reactivity. Attempts to define heavy metals by atomic weight or mass were equally ambiguous, and the term was even applied to semimetals such as arsenic (As) [5]. Importantly, the International Union of Pure and Applied Chemistry, the gold standard of chemical nomenclature, does not formally recognize “heavy metals,” highlighting its imprecision and the misleading impression of a standardized classification.

The classification of a toxic factor, according to the Glossary of Terms Used in Toxicology, 2nd edition [6], is defined as “able to cause injury to living organisms as a result of physicochemical interaction,” whereas toxicity is defined as: (i) The capacity to cause injury to a living organism, taking into account the quantity of substance administered or absorbed, the route and timing of administration (single or repeated doses), the type and severity of injury, the time required to produce the injury, the nature of the organism(s) affected, and other relevant conditions; (ii) Adverse effects of a substance on a living organism as defined in point 1; and (iii) A measure of the incompatibility of a substance with life, which may be expressed as the reciprocal of the absolute value of the median lethal dose (1/LD₅₀) or concentration (1/LC₅₀).

To provide clarity, in this perspective article we adopt the term “toxic and potentially toxic elements”, which we further categorize into non-essential and essential elements, the latter being necessary for normal human physiological function. The toxicity of toxic and potentially toxic elements, such as cadmium (Cd) and lead (Pb), depends on their concentration, chemical form, bioavailability, and bioaccessibility (Table 1). For example, human exposure to Pb through tetraethyl-lead in gasoline or lead-based paint is well documented and poses a clear health risk, whereas lead-acid batteries do not present a direct exposure risk but may contribute to environmental contamination [7].

**Table 1:** Possible effects of intoxication and absorption routes of toxic and potentially toxic elements in humans

Toxic and potentially toxic elements — non-essential		
Nomenclature	Possible effects of intoxication on human physiology	Routes of absorption
Aluminum (Al)	May affect the nervous system, blood-brain barrier, bones, and brain; possible link to Alzheimer’s disease. Can cause dermatitis, digestive issues, and vomiting.	Absorbed via oral and inhalation routes, with minimal dermal absorption.
Antimony (Sb)	Can cause epiglottis irritation, increased alveolar and bronchiolar macrophages, reduced pulmonary clearance, pulmonary fibrosis, myocardial damage, nausea, and vomiting.	Poorly absorbed; can deposit in the respiratory tract and enter the body via the gastrointestinal tract, bloodstream, or lymphatic system, subsequently reaching the thoracic lymph nodes. Not metabolized; excreted in urine and feces.
Arsenic (As)	May cause gastrointestinal symptoms, weakness, shock, unconsciousness, dermatitis, neuropathy, respiratory irritation, and skin hyperpigmentation. Potential occupational carcinogen.	Can be absorbed via inhalation, ingestion, dermal, or ocular exposure; both organic and inorganic forms are excreted in urine.
Barium (Ba)	Can cause hypokalemia, vomiting, abdominal cramps, diarrhea, electrocardiogram abnormalities, muscle weakness, and paralysis.	Can be absorbed orally, by inhalation, or through dermal contact; crosses the placenta and can be transferred via breast milk. Mostly excreted in feces and urine, with small amounts accumulating in bones and teeth.
Beryllium (Be)	May cause cough, sore throat, and shortness of breath, as well as anorexia, weight loss, fatigue, chest pain, cyanosis, pulmonary failure, eye irritation, dermatitis, and digital clubbing. Potential occupational carcinogen.	Can accumulate in the lungs with minimal dermal absorption. Absorbed by inhalation, ingestion, or ocular contact; unabsorbed portions are excreted in feces.
Bismuth (Bi)	Hepatotoxic; may cause stomatitis, oral mucosa hyperpigmentation, renal dysfunction, myoclonus, and encephalopathy.	Minimally to moderately absorbed via inhalation, oral, or topical routes; gastrointestinal absorption is possible (salt forms). Retained in the kidneys and mostly excreted in urine.
Boron (B)	May irritate the eyes and respiratory tract, causing dryness, nosebleeds, and sore throat. It can affect seminal fluid and sperm and may be fatal if ingested at high levels. May also induce gastrointestinal effects, hemorrhagic damage, vascular blockages, and cardiopulmonary hypotension.	Absorbed orally and via inhalation; minimal dermal absorption; mainly excreted in urine.
Cadmium (Cd)	Pulmonary edema, dyspnea, cough, chest pain, headache, chills, muscle pain, nausea, vomiting, diarrhea, anosmia, emphysema, proteinuria, and mild anemia. Potential occupational carcinogen.	Absorbed by ingestion or inhalation; can persist in the kidneys and liver for years and is slowly excreted in urine and feces.
Germanium (Ge)	Acts as a neurotoxin, hepatotoxin, and nephrotoxin.	Absorbed orally and via inhalation; mainly excreted in urine with some fecal excretion.
Gold (Au)	May cause chrysiasis, ocular effects, dermatitis, stomatitis, renal changes, eosinophilia, cytopenias, and aplastic anemia.	Can be absorbed via inhalation, dermal contact, acupuncture, piercings, medical prostheses, dental procedures, or gold therapy for rheumatoid arthritis. Excreted via skin, hair, nails, bile, urine, and feces.
Lead (Pb)	May cause cough, metallic taste, abdominal pain, headache, confusion, drowsiness, seizures, anorexia, fatigue, facial pallor, weight loss, malnutrition, constipation, colic, anemia, gingival lead line, tremor, wrist and ankle drop, encephalopathy, kidney disease, hypertension, and, in severe cases, coma and death.	Can be absorbed via the gastrointestinal or pulmonary routes, binding mainly to erythrocyte proteins and partially to plasma before reaching target organs.
Lithium (Li)	Bradycardia, T-wave changes, sick sinus syndrome, confusion, memory problems, tremors, hyperreflexia, clonus, slurred speech, ataxia, stupor, delirium, coma, rare seizures, nephrogenic diabetes insipidus, leukocytosis, aplastic anemia, gastrointestinal symptoms, thyroid disorders, shock, weakness, irritation and burns of the eyes and skin, and respiratory symptoms including cough, difficulty breathing, and sore throat.	Readily absorbed in the gastrointestinal tract, minimally bound to plasma proteins, not metabolized, and excreted unchanged in urine.
Mercury (Hg)	Acute: pneumonitis, tremors, and tissue damage. Chronic: lymphocytic aneuploidy, dysmenorrhea, neurological and psychiatric effects, kidney dysfunction, upper airways inflammation, anxiety, emotional lability, memory and sleep disturbances, fatigue, movement and speech disorders, gum discoloration, and anorexia.	Absorbed via inhalation, dermally (slowly), or through ocular contact; gastrointestinal absorption of elemental mercury is minimal.
Nickel (Ni)	Can cause dermatitis, headache, gastrointestinal and respiratory symptoms, pulmonary fibrosis, cardiovascular diseases, cancer, and epigenetic effects.	The toxic and carcinogenic effects depend on absorption, physicochemical properties, dose, duration of exposure, and route of exposure. It can enter the body via inhalation, ingestion, or dermal contact, depending on its chemical form.
Scandium (Sc)	Induces tissue damage and fibrosis.	Retained in the bladder, liver, and bones after intravenous administration; data on oral, dermal, and intestinal absorption are insufficient.
Silicon (Si)	Cough; redness and irritation of the eyes, skin, and upper respiratory tract.	Absorbed via inhalation, ingestion, or ocular and dermal contact.
Silver (Ag)	May cause abdominal pain, ocular and dermal burns, deposits in the conjunctiva, cornea, and central nervous system, argyria, and mild allergic reactions.	Can be absorbed via inhalation, ingestion, and dermal absorption.
Strontium (Sr)	May cause strontium rickets, altered parathyroid hormone, and rare anaphylactic or tachycardia reactions (limited data).	Absorbed as an aerosol; distribution after inhalation is likely similar to that after ingestion. Accumulates in the skeleton and can be transferred to the fetus during pregnancy.
Sulfur (S)	Causes eye redness and burning, cough, sore throat, blurred vision, and diarrhea.	Can be absorbed through the skin and detected in blood and urine.
Tellurium (Te)	Causes garlic breath, changes in sweating, dry mouth, drowsiness, gastrointestinal symptoms, dermatitis, headache, eye pain and redness, and constipation.	Can be absorbed through the skin (soluble forms), by oral ingestion, or via inhalation. Can be excreted in urine, feces, and bile.
Titanium (Ti)	May induce Yellow Nail Syndrome.	Poorly absorbed orally; may cross the placenta; possibly excreted in urine.
Tin (Sn)	May cause abdominal pain, anemia, and liver or kidney dysfunction (inorganic compounds).	Poorly absorbed via oral or inhalation routes; limited dermal absorption; excreted in feces and urine.
Tungsten (W)	May cause pulmonary fibrosis, sensory and memory deficits, increased mortality, and ocular or dermal irritation.	Absorbed via inhalation, oral, and dermal routes; excreted in urine or feces; may accumulate in bones, nails, or hair; crosses the placental barrier.
Vanadium (V)	May cause headache, central nervous system depression, gastrointestinal symptoms, eye irritation, respiratory effects, peripheral vasoconstriction, and dermatitis.	Poorly absorbed through the skin and gastrointestinal tract; primarily absorbed via inhalation, widely distributed with the highest concentrations in adipose tissue, and excreted in urine and feces.
Zirconium (Zr)	May cause redness and irritation of the eyes, and cutaneous or pulmonary granulomas.	Absorbed via inhalation or through ocular or dermal contact.
**Potentially toxic elements — essential **		
Calcium (Ca)	May cause gastrointestinal, ocular, dermal, and respiratory irritation, including burns, pain, vomiting, cough, and shortness of breath.	Absorption decreases with increasing intake; absorbed through active and passive intestinal transport; excreted in urine.
Chromium (Cr)	May cause respiratory, ocular, and dermal irritation, pulmonary fibrosis, nephrotoxicity, carcinogenicity, asthma, dermal burns, and sensitization.	Can be absorbed via inhalation, ingestion, or dermal and ocular contact. Excreted mainly in urine as Cr(III), and to a lesser extent via bile, breast milk, saliva, and sweat.
Cobalt (Co)	Causes cardiomyopathy, neuropathy, vision loss, and chronic respiratory disease; cobalt alone (without tungsten carbide) is potentially carcinogenic.	Distribution is influenced by plasma proteins; uptake involves the P2X7 receptor (P2X purinergic receptor 7) and divalent metal transporter 1. Gastrointestinal absorption averages 25%, with high interindividual variability (5–97%).
Copper (Cu)	Causes acute hepatotoxicity and gastrointestinal distress and may lead to cardiovascular collapse, coma, death, shock, renal failure, rhabdomyolysis, and severe hemolytic anemia.	Absorption decreases with higher intake; once systemically absorbed, it is retained in the respiratory system, and mainly eliminated via bile.
Iron (Fe)	May cause oxidative stress, coagulopathy, liver and kidney damage, and cardiomyopathy.	Absorbed in the duodenum and proximal jejunum, depending on its physical state.
Magnesium (Mg)	May cause electrolyte imbalances, cardiovascular and respiratory effects, gastrointestinal symptoms, flushing, and irritation of the ocular, dermal, and respiratory tissues.	Absorbed mainly via passive intestinal transport; primarily excreted by the kidneys.
Manganese (Mn)	May cause tremors, muscle spasms, tinnitus, hearing loss, unsteadiness, mood and cognitive changes, insomnia, headache, anorexia, and reduced motor coordination.	Absorbed in the small intestine via active transport and diffusion. Most of it binds to transferrin, albumin, and alpha-2-macroglobulin, and is subsequently taken up by the liver and other tissues through a mechanism not yet fully understood.
Molybdenum (Mo)	May cause liver damage and alter levels of prolactin, sperm concentration, testosterone, and other androgens.	Absorbed orally or via inhalation; minimal dermal uptake (~0.2%). Concentrates in the liver and kidneys, undergoes minor redox reactions, and is primarily excreted in urine.
Phosphorus (P)	May cause chronic kidney disease and increase the risk of cardiovascular disease and mortality.	Absorbed in the small intestine, partially via active transport; excreted in urine.
Potassium (K)	May cause dermal burns, hyperkalemia, nausea, diarrhea, abdominal pain, and vomiting. May also trigger hypersensitivity reactions and, with intravenous administration, phlebitis, extravasation, and local irritation.	Absorbed orally in the small intestine; primarily excreted in urine and feces, with minimal excretion in sweat.
Sulfur (S)	May cause redness and burning of the skin and eyes, cough, sore throat, blurred vision, and diarrhea.	Can be absorbed dermally and detected in blood and urine.
Selenium (Se)	May cause gastrointestinal symptoms; respiratory irritation including bronchial spasms and coughing; irritation of the eyes, skin, nose, and throat; headache; fever; dyspnea; metallic taste; garlic breath; dermatitis; and dermal burns.	Can be absorbed via inhalation and ingestion, as well as contact with skin and eyes.
Sodium (Na)	May cause respiratory irritation; dermal, ocular, and oral burns; vision loss; and shock or collapse.	Absorbed in the distal small intestine and colon; primarily excreted in urine.
Zinc (Zn)	May cause nausea, vomiting, epigastric pain, and fatigue.	Absorbed in the small intestine via transporter-mediated mechanisms, and excreted in urine, hair, sweat, dermal secretions, and through intestinal and biliary pathways.

References: [32–46]. %: percentage

The degradation of water quality resulting from the release of toxic and potentially toxic elements is one of the earliest and most significant consequences of mining activities. Water contamination by toxic and potentially toxic elements can occur through two main pathways: point sources and diffuse sources. Point sources refer to the direct discharge of effluents containing toxic and potentially toxic elements [e.g., aluminum (Al), mercury (Hg), and Pb], into surface water bodies such as rivers, lakes, and ponds from an identified outfall. In contrast, diffuse sources involve the transport of contaminants through surface runoff from rainfall, which carries soil particles and toxic residues into aquatic ecosystems [8]. Due to the high water demand of industrial operations, which in itself already constitutes an environmental concern, such facilities are often located near rivers, lakes, or reservoirs [3, 8].

Human exposure to toxic and potentially toxic elements can occur via multiple routes, including direct contact with contaminated water, air, or soil, as well as through the consumption of contaminated food, such as fish and vegetables grown in affected areas. In addition, essential elements that are fundamental to biological processes, such as cobalt (Co), copper (Cu), iron (Fe), manganese (Mn), nickel (Ni), and zinc (Zn), may become toxic when present in excess. The bioaccumulation of these metals in the human body can lead to significant levels of toxicity, with clinical manifestations depending on the chemical element, concentration, and duration of exposure (Table 1). Even at subclinical levels, chronic exposure poses health risks. The main organs and systems affected include the central nervous system, kidneys, liver, immune system, and cardiovascular system [9].

The populations most affected by water contamination from toxic and potentially toxic elements are those living along the banks of rivers and lakes, known as riparian populations, as well as communities residing in forested areas or near mining operations, such as Indigenous and Quilombola peoples. These populations, in addition to facing greater socio-environmental vulnerability, are among the highest consumers of fish worldwide, making them particularly susceptible to chronic exposure to toxic and potentially toxic elements through the food chain [10, 11].

The Brazilian context highlights the severity of the problem. According to a survey by the Amazon Network of Georeferenced Socio-Environmental Information (RAISG of the Portuguese *Rede Amazônica de Informação Socioambiental Georreferenciada*), there are at least 453 illegal mining sites spread across the Brazilian Amazon [12], many of which overlap with Indigenous territories and protected areas. In a cross-sectional study conducted by Basta et al. (2021), Hg exposure levels were assessed in 200 Indigenous individuals from three different Brazilian communities. The average prevalence of Hg levels above the recommended limit was 57.9% among the villages assessed. The most alarming case involved a 10-year-old child who presented a blood Hg concentration of 23.9 µg·L⁻¹, illustrating the critical impact of environmental contamination on vulnerable population groups [10].

## The Xikrin do Cateté Territory: Environmental Pressure, Mining Activities, and Socio-Cultural Impacts

A recent situation involving the Indigenous peoples of the Xikrin do Cateté Tribe, located in the State of Pará, Brazil, has gained attention in both national and international media. This territory is home to four distinct Indigenous groups, Guarani, Guarani Mbya, Mebengôkre (Kayapó), and Xikrin Mebengôkre, comprising a total population of 1,737 inhabitants, with no overlap with other environmental conservation units.

Although traditionally defined as hunters, the Xikrin also rely on agriculture and gathering. Hunting remains fundamental to their diet and culture, involving species such as tapir, peccary, deer, paca, and agouti. Fishing, particularly during the winter and summer periods, constitutes another important food source. However, both hunting and fishing are increasingly affected by environmental degradation, including siltation and river contamination. The headwaters of these rivers, located outside the boundaries of their demarcated lands, are being impacted by mining and deforestation activities [13].

According to data from the National Institute for Space Research (INPE of the Portuguese *Instituto Nacional de Pesquisas Espaciais*), cumulative deforestation in this region reached 4,470 hectares by the year 2000 and totaled 7,448 hectares by 2024. Additionally, INPE recorded 446 fire outbreaks in the past year alone, highlighting the growing environmental pressure on the territory. The Xikrin do Cateté Indigenous Land is continuously affected by mining activities located downstream of its two main rivers, the Itacaiúnas and the Cateté. Currently, there are at least eight ongoing legal proceedings against mining companies operating in the region: five related to Cu extraction, two to gold (Au), and one to Ni [14].

The Xikrin do Cateté territory is surrounded by 12 mineral extraction complexes, all operated by the company Vale S.A., with particular emphasis on the “Onça-Puma” project, dedicated to Ni extraction and situated along the banks of the Cateté river. Intensive mining activity has caused significant environmental degradation, particularly the contamination of soil and, most critically, the rivers that sustain the livelihood and culture of the local Indigenous peoples.

Although mining activities may contribute to Hg exposure in Indigenous populations, they should not be considered an isolated or exclusive source. In the Amazon region, for example, other extractive activities, such as oil exploitation, have also been associated with environmental contamination and human exposure to toxic and potentially toxic elements, including As, Cd, and Pb, in addition to Hg [15].

## Biomonitoring Evidence: Analytical Methods, Exposure Profiles, and Public Health Implications

The consequences of this contamination were revealed through studies conducted by researchers from the State of Pará, Brazil, who analyzed biological samples from 732 Indigenous individuals residing in the region. The sampling was designed to achieve a statistical error margin of ± 3% for a total target population of approximately 1,600 individuals. The analyses were performed in May 2024 using specialized laboratory services (Biominerais, Campinas, São Paulo, Brazil) and a trained field team deployed to 21 villages within the Xikrin Indigenous Territory. All data analyzed in this study are publicly available as part of the documentation associated with Civil Public Action n° 1,001,462 − 67.2025.4.01.3901 [13]. However, access to raw analytical data, detailed laboratory protocols, and quality assurance/quality control (QA/QC) procedures is restricted, as these materials are embedded within judicial and technical reports submitted under legal confidentiality and procedural constraints. Consequently, granular methodological details and primary laboratory datasets cannot be independently retrieved or reproduced outside the scope of the ongoing legal proceedings.

Hair samples were collected and analyzed using hair mineralogram (mineralography), allowing the assessment of elemental profiles, including both toxic and potentially toxic elements and essential elements in excess. Chemical element quantification was performed using Inductively Coupled Plasma Atomic Emission Spectrometry (ICP-AES, also known as ICP-OES) and Graphite Furnace Atomic Absorption Spectrometry (GFAAS). The GFAAS reference value was applied only for selenium (Se), whereas all other elements were interpreted according to ICP-AES reference parameters.

Reference values were provided by the researchers and technical staff of the BioMinerais Laboratory, who indicated that these values were informed by World Health Organization (WHO) guidelines. These reference values are documented in the public records of the ongoing Civil Public Action; however, we do not have independent technical or scientific confirmation that the WHO has formally established reference values for all elements analyzed. The interpretation of concentrations of all toxic and potentially toxic elements, including both essential and non-essential elements, was guided by the WHO chemical safety framework as a recognized public-health reference, while acknowledging the limitations of applying these benchmarks in the absence of formally established values for hair biomonitoring.

In a systematic review assessing reference intervals for elements in human hair, the reference values and reference intervals were established in accordance with the criteria of the International Federation of Clinical Chemistry (IFCC). According to the IFCC, a reference interval is defined as the prediction interval within which 95% of the values obtained from a defined reference population are expected to fall. The review identified 52 published reports; however, only five studies met both the IFCC methodological standards and the predefined inclusion criteria. Furthermore, the authors highlighted the lack of standardized procedures for establishing reference values for minerals in hair and emphasized the highly individualized nature of these measurements, which may vary according to sex, age, and race [16]. Subsequently, although several studies have investigated hair as a biomarker of Hg exposure among Indigenous populations, such as the Yanomami, Munduruku, and the Indigenous peoples of the Kayapó Territory (Fresco River region), a comprehensive table of reference values for minerals in hair applicable to the entire Brazilian Indigenous population has not yet been established. This absence of standardized reference values substantially limits the interpretability and comparability of the findings [17–19].

A systematic review evaluating hair as a biomarker of long-term Hg exposure reported that Hg levels in Amazonian populations frequently exceed the limits recommended by the WHO. According to WHO guidance, typical background concentrations range from 1 to 2 µg·g⁻¹, while levels above 10 µg·g⁻¹ are considered indicative of high exposure among daily fish consumers. In the general Amazonian population, mean Hg concentrations reached 29.59 µg·g⁻¹, with values ranging from 0.73 to 97.44 µg·g⁻¹. Elevated concentrations were also observed among Indigenous populations, with a mean value of 6.95 µg·g⁻¹ (range: 4.90–8.37 µg·g⁻¹). Importantly, the review highlighted that even populations classified as “unexposed” may still be at risk of adverse health effects associated with Hg exposure. The authors further noted that a substantial proportion of the available studies were conducted in the State of Pará, Brazil, consistent with the high density of legal and illegal Au mining activities in the region, particularly within the Tapajós basin. Additionally, most investigations were carried out along major river systems, including the Negro, Tapajós, and Madeira rivers [20].

**Fig. 1:**
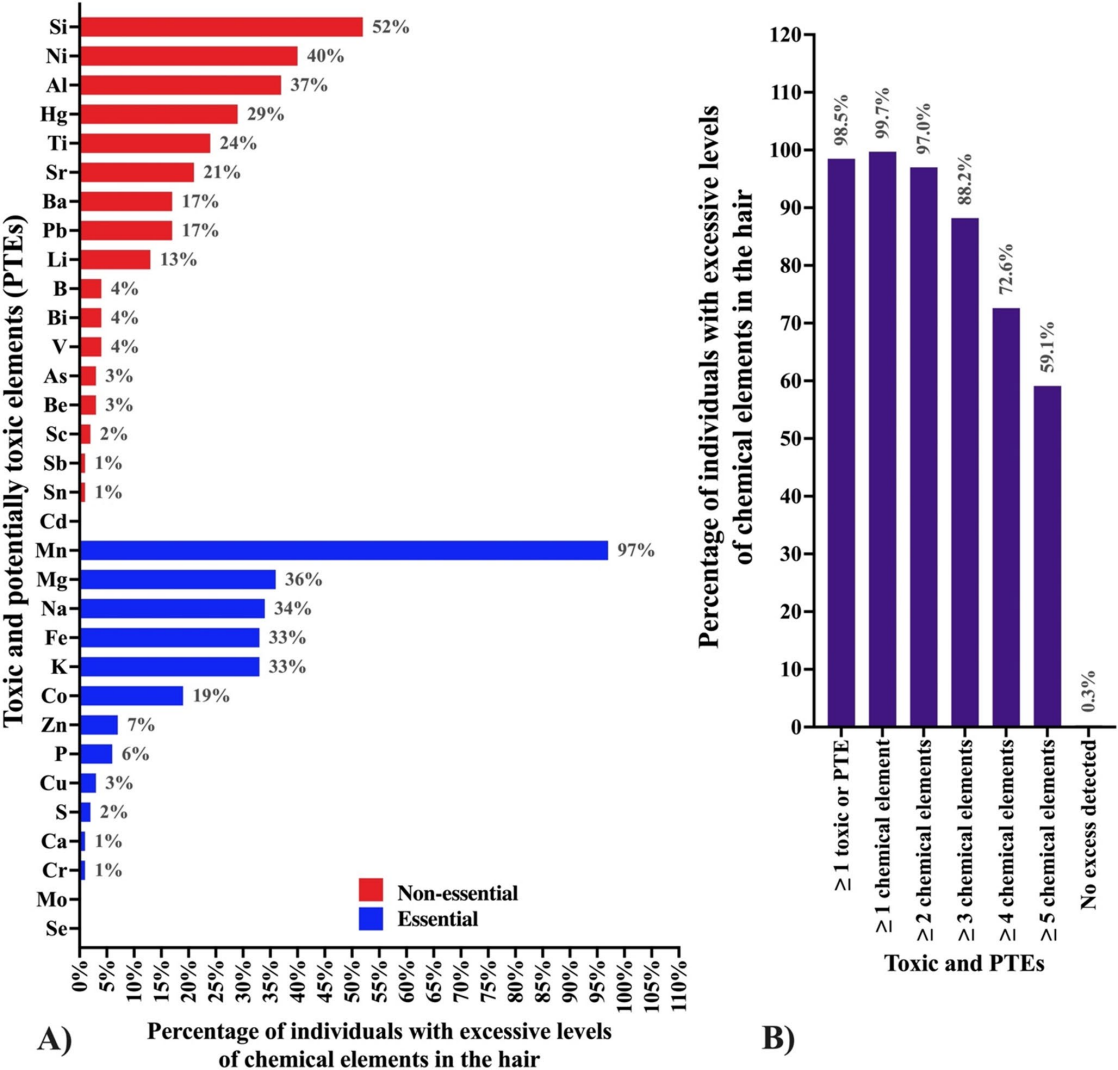
Description of toxic and potentially toxic elements (PTEs) found in Indigenous peoples from the Xikrin Village of the Cateté Tribe, located in the State of Pará, Brazil. **A** Percentage of Indigenous individuals with elevated levels of toxic and PTEs classified as essential and non-essential. **B** Grouping of the number of Indigenous individuals according to the number of toxic and PTEs at elevated levels. Ag: Silver, Al: Aluminum, As: Arsenic, Au: Gold, Ba: Barium, Be: Beryllium, Bi: Bismuth, B: Boron, Ca: Calcium, Cd: Cadmium, Co: Cobalt, Cr: Chromium, Cu: Copper, Fe: Iron, Ge: Germanium, Hg: Mercury, K: Potassium, Li: Lithium, Mg: Magnesium, Mn: Manganese, Mo: Molybdenum, Na: Sodium, Nb: Niobium, Ni: Nickel, P: Phosphorus, Pb: Lead, Sc: Scandium, Se: Selenium, Si: Silicon, Sn: Tin, Sr: Strontium, S: Sulfur, Te: Tellurium, Ti: Titanium, V: Vanadium, W: Tungsten, Zr: Zirconium, %: percentage

In the documentation associated with Civil Public Action n° 1,001,462 − 67.2025.4.01.3901, of the 732 Indigenous individuals evaluated, 709 (98.5%) exhibited concentrations of one or more toxic or potentially toxic elements exceeding the reference values (Table 2). A total of 32 chemical elements were detected, of which 22 metals and semimetals exceeded the reference values adopted in this study, which were defined according to the reference thresholds used by the analytical laboratory and those reported in the public documentation associated with the Civil Public Action. Among these, seven elements are recognized for their toxicological relevance due to absence of an essential physiological role in the human body and their inherent toxicity. These include six metals, Al (detected in 36.5% of individuals), Hg (28.0%), titanium (Ti; 24.0%), Pb (17.0%), barium (Ba; 17.0%), and beryllium (Be) (3.3%), and one semimetal, As (2.8%) [13] (Fig. 1A and B). Notably, Hg and As are of particular concern, as no safe exposure threshold has been established for either element, and adverse health effects have been reported even at very low levels of exposure. In this study, an element was classified as excessive when its concentration in hair exceeded the reference range established by the BioMinerais Laboratory. Therefore, the values in Table 2 refer to the number of elements above reference concentrations for each participant, not the number of elements detected in the sample.

**Table 2:** Number and percentage of Indigenous participants presenting concentrations of toxic or potentially toxic elements above reference values in hair samples

Number of excessive elements in the hair	*N*	%
≥ 1 toxic element	709	98.5%
≥ 1 chemical element	718	99.7%
≥ 2 chemical elements	698	97.0%
≥ 3 chemical elements	635	88.2%
≥ 4 chemical elements	523	72.6%
≥ 5 chemical elements	404	59.1%
No excess detected (essential or non-essential chemical element)	2	0.3%

%: percentage, ≥: greater than or equal to, N: number of individuals. A total of 720 Indigenous were evaluated in the study

Reference values were provided by the researchers and technical staff of the BioMinerais Laboratory, who indicated that these values were informed by World Health Organization (WHO) guidelines. These reference values are documented in the public records of the ongoing Civil Public Action; however, we do not have independent technical or scientific confirmation that the WHO has formally established reference values for all elements analyzed

Importantly, among the 121 children analyzed, all were found to have one or more metals present in their bodies at excessive and hazardous levels for human health [21, 22]. While the report confirms the presence of hazardous concentrations in all analyzed children, individual-level data on specific metal concentrations are not publicly available, as the report provides only aggregate results. According to the Federal University of Pará (UFPA of the Portuguese *Universidade Federal Pará*) technical report, contamination by toxic and potentially toxic elements among the Xikrin Indigenous people is virtually universal and constitutes a public health emergency, demanding immediate attention from health authorities.

**Fig. 2:**
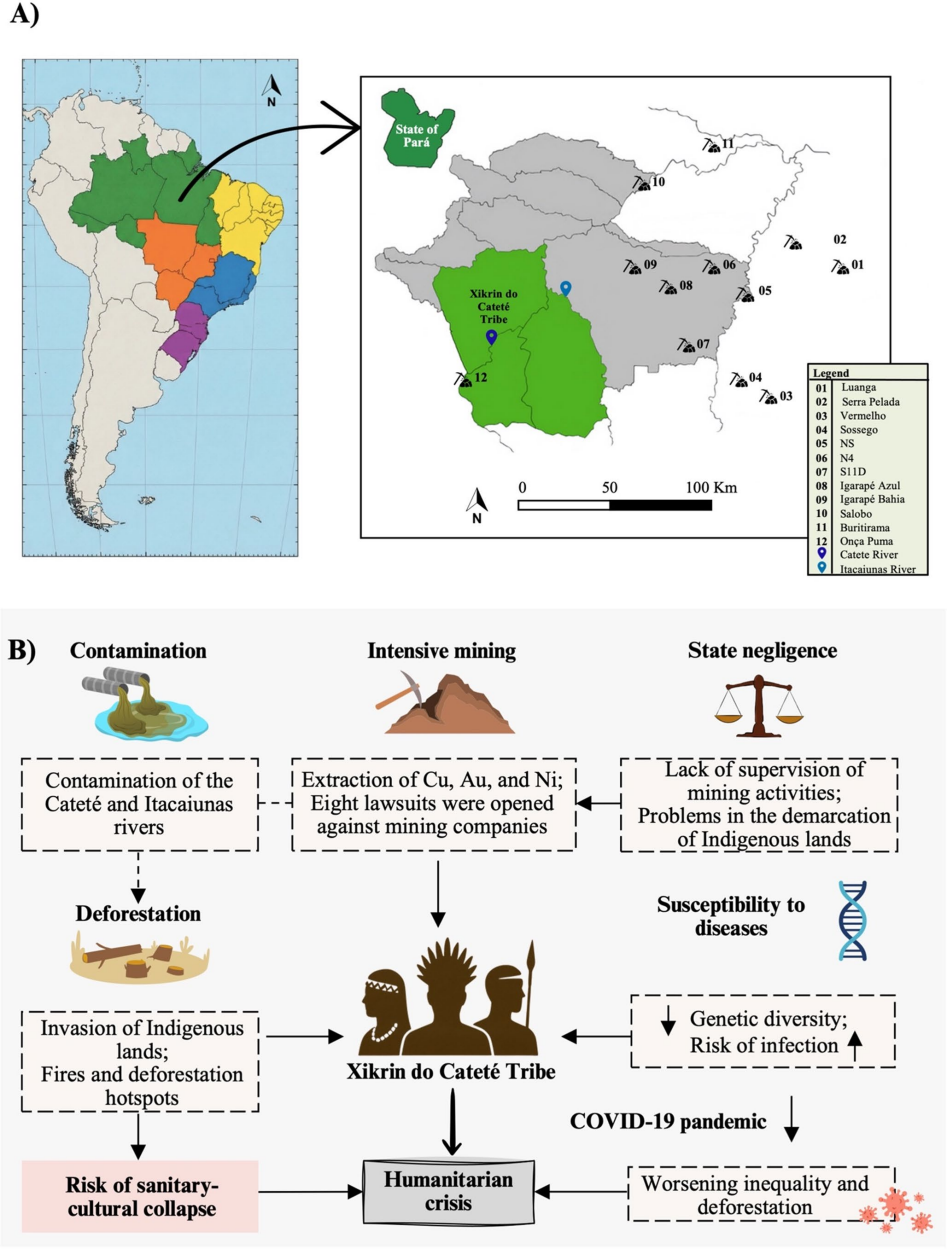
General characterization of the findings presented in the study on the presence of toxic and potentially toxic elements in Indigenous individuals from the Xikrin do Cateté Tribe, located in the State of Pará, Brazil. **A** Map showing the location of the Xikrin do Cateté Tribe in Brazil and the presence of mining sites numbered from 1 to 12. All analyzed data are publicly available in the documentation of Civil Public Action n° 1,001,462 − 67.2025.4.01.3901. **B** Description of the main factors associated with the humanitarian crisis caused by contamination with toxic and potentially toxic elements in Indigenous individuals from the Xikrin do Cateté Tribe. Au: gold, COVID-19: coronavirus disease 2019, Cu: copper, Ni: nickel

**Fig. 3:**
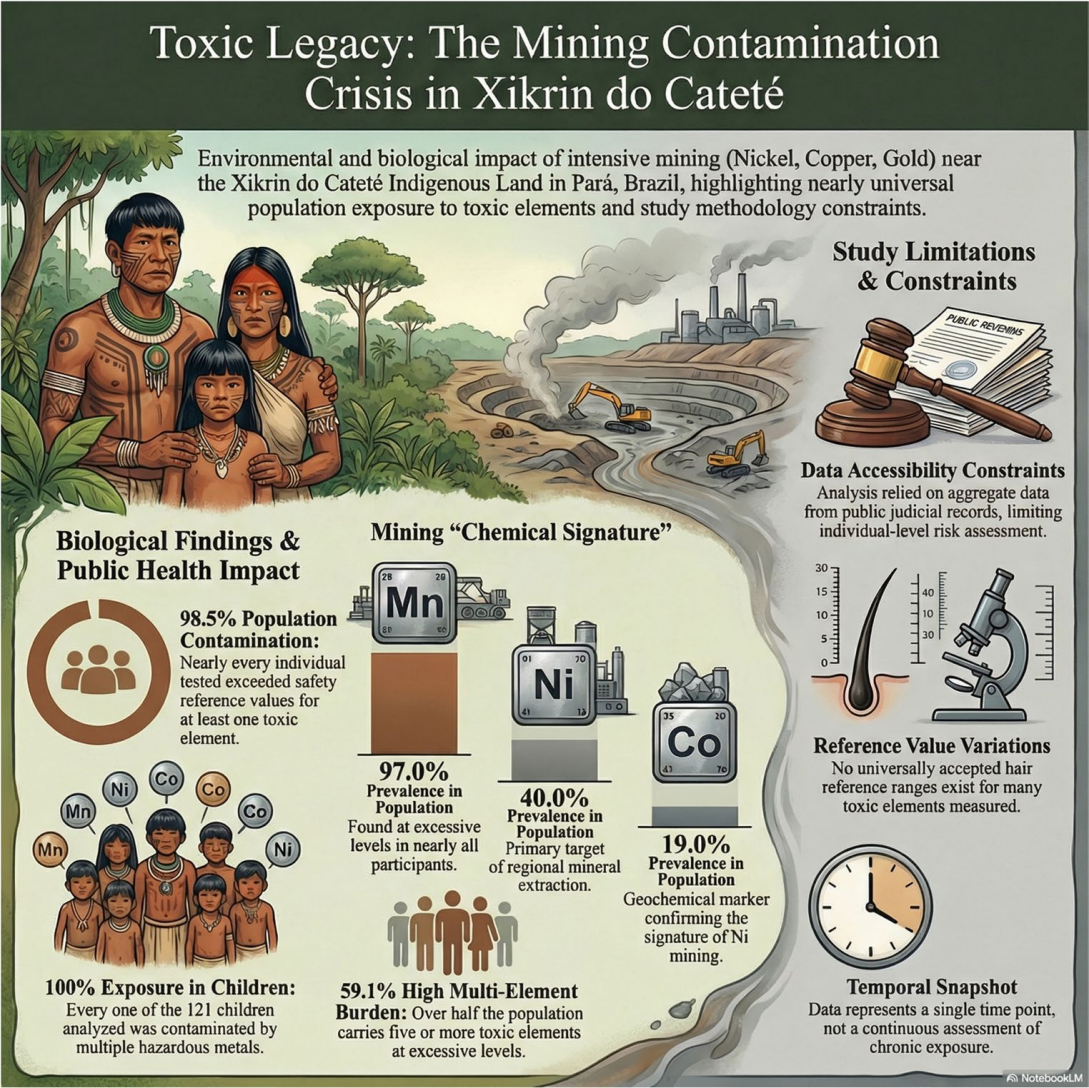
Conceptual summary of mining-associated environmental contamination and multi-element exposure profile observed in individuals from the Xikrin do Cateté Indigenous Territory, highlighting prevalence of cobalt (Co), manganese (Mn), and nickel (Ni), and key public health implications. NotebookLM (Google LLC) was used under an institutional license provided by the University of São Francisco (USF of the Portuguese Universidade São Francisco) through Google Workspace for Education. This license authorizes faculty and students to use the tool for academic and research purposes. The platform supported the organization and analysis of academic materials, and the figure included in this work was generated using the application under this licensed access. Data usage followed institutional policies for privacy, security, and responsible use of digital tools, %: percentage

The most prevalent toxic or potentially toxic elements in the analyzed sample, for example, included Mn, present in 97.0% of individuals, followed by Ni at 40.0%, Fe at 32.5%, and Co at 19.0%. The latter deserves special attention, as Co is considered a characteristic marker of Ni mining operations, functioning as a veritable “chemical signature” of extractive activities in the region. Even more alarmingly, a significant accumulation of toxic chemical elements was identified in an Indigenous child only one year old. Among the contaminants detected at elevated levels were Al, Ba, Be, Cd, Pb, and Ti, underscoring the severity and extent of environmental exposure endured by highly vulnerable populations [13]. The exact concentrations of toxic or potentially toxic elements detected in the hair of two Indigenous children, aged 1 and 4 years, respectively, are presented in Table 3, along with the possible clinical and systemic repercussions associated with chronic and acute exposure, both in the short and long term. A general overview of the findings is presented in Fig. 2 and the Graphical Abstract. Figure 3 presents a conceptual overview of mining-related environmental contamination and the high prevalence of toxic and potentially toxic elements detected in individuals from the Xikrin do Cateté Indigenous Territory.

**Table 3:** Concentration of toxic and potentially toxic elements in two Indigenous children of the Xikrin do Cateté Tribe and reference values

Chemical elements	Concentration (mg·kg⁻¹)		Reference range for non-exposed populations*
	Indigenous 1^a^	Indigenous 2^b^	
Toxic and potentially toxic elements — non-essential			
Aluminum (Al)	**47.2**	**39.1**	1.0–9.0
Antimony (Sb)	< 0.02	< 0.02	0.03–0.9
Arsenic (As)	< 0.01	< 0.01	0.02–0.8
Barium (Ba)	**11.8**	< 0.07	1.0–2.5
Beryllium (Be)	0.02	< 0.01	0.01–0.05
Bismuth (Bi)	< 0.01	< 0.01	0.05–0.1
Boron (B)	< 0.8	< 0.8	1.0–3.5
Cadmium (Cd)	0.36	0.07	0.02–0.8
Germanium (Ge)	< 0.01	< 0.01	0.1–0.4
Gold (Au)	< 0.019	< 0.019	0.019–0.25
Lead (Pb)	1.5	1.4	0.5–2.0
Lithium (Li)	0.005	0.015	0.010–0.025
Mercury (Hg)	< 0.03	< 0.03	0.05–0.6
Nickel (Ni)	**1.17**	**1.68**	0.1–0.7
Scandium (Sc)	< 0.005	< 0.005	0.018–0.023
Silicon (Si)	**32.1**	**72.5**	10.9–27.9
Silver (Ag)	< 0.04	< 0.04	0.2–1.0
Strontium (Sr)	10.8	7.4	0.5–11.0
Tellurium (Te)	< 2	< 2	* – *
Titanium (Ti)	**1**	0.8	0.04–0.8
Tin (Sn)	< 0.01	0.03	0.3–1.6
Tungsten (W)	< 0.2	< 0.2	0.3–2.4
Vanadium (V)	**0.5**	**0.65**	0.1–0.4
Zirconium (Zr)	< 0.02	< 0.02	0.05–1.0
Potentially toxic elements — essential			
Calcium (Ca)	1,144	1,083	280–2,500
Chromium (Cr)	0.2	0.5	0.3–1.4
Cobalt (Co)	**0.49**	0.19	0.06–0.3
Copper (Cu)	12.6	9	8–45.0
Iron (Fe)	46	**93**	10–47
Magnesium (Mg)	117	194	34–200
Manganese (Mn)	**9.08**	**8.07**	0.24–1.00
Molybdenum (Mo)	0.1	0.02	0.025–0.20
Phosphorus (P)	159	148	120–209
Potassium (K)	67	71	5–165
Sulfur (S)	36,359	37,053	21,200–45,000
Selenium (Se)	0.18	0.28	0.18–2.5
Sodium (Na)	185	302	28–400
Zinc (Zn)	218	101	125–240

^a^: One-year-old Indigenous child, ^b^: Four-year-old Indigenous child

* - *: Unknown concentrations, mg·kg⁻¹: milligrams per kilogram

*: Reference values were provided by the researchers and technical staff of the BioMinerais Laboratory, who indicated that these values were informed by World Health Organization (WHO) guidelines. These reference values are documented in the public records of the ongoing Civil Public Action; however, we do not have independent technical or scientific confirmation that the WHO has formally established reference values for all elements analyzed. These values were used exclusively for descriptive comparison and do not represent toxicological thresholds

The bold values indicate concentrations that exceed the reference values adopted for interpretation. These reference values were those used by the analytical laboratory and explicitly reported both through direct communication with the laboratory and in the public documentation associated with Civil Public Action n° 1,001,462 − 67.2025.4.01.3901

The inclusion of data from two children in Table 3 does not aim to provide statistical inference or represent outliers. Rather, these cases were the only individual-level examples made publicly available in the official technical report, and their presentation serves an illustrative and contextual purpose. Importantly, these children were highlighted in the original report because they exemplify the multi-element exposure profile observed at the population level, characterized by the concurrent elevation of several toxic and potentially toxic elements rather than isolated single-metal exposure. This pattern is documented in the aggregate results, which show that 98.5% of the analyzed population presented at least one element above reference values and that multiple elements were simultaneously elevated across the cohort.

According to the report, these two children exhibited markedly elevated concentrations of several toxic elements, including Al, Ba, Pb, and other potentially toxic elements known to exert neurotoxic, nephrotoxic, and hematological effects (Tables 1 and 3). Their inclusion aimed to demonstrate how bioaccumulation of toxic elements occurs even at early stages of life, reinforcing the hypothesis of prenatal exposure and possible transmission through breastfeeding, as previously suggested in related sections of the report. From a scientific standpoint, the inclusion of these two cases functions as an illustrative validation of the analytical process and as an ethical compromise between transparency and confidentiality. Their presentation provides a clear visual and numerical reference for the population-wide trends while respecting the ethical imperatives of anonymity and the protection of vulnerable groups.

It is important to acknowledge that children differ substantially from adults in toxicokinetics, including absorption, distribution, metabolism, and excretion of chemical elements, and that sex-related biological differences may further influence exposure profiles and biological responses. These factors are particularly relevant in the context of toxic and potentially toxic elements, for which age- and sex-specific variability may affect internal dose and health effects. However, the present study was limited by the exclusive availability of aggregated data derived from public documentation, which precluded stratified analyses according to age, sex, or other individual-level characteristics. Consequently, no age- or sex-specific inferences could be performed. In light of these constraints, the individual cases presented are intended solely for illustrative purposes, and no mechanistic or causal conclusions were extrapolated from these examples.

Table 1 summarizes potential toxicological effects and absorption routes. These effects represent possible outcomes of high or prolonged exposure and are not directly inferred from the concentrations observed in this study. The levels detected in the two children cannot be directly compared with toxic doses, which depend on the chemical form, exposure route, and cumulative burden. Therefore, Table 1 serves an informative and contextual purpose rather than a risk-classification tool.

Several elements that exceeded the reference ranges are consistent with the mineralogical profile and Ni-focused (Nickel-focused) mining activities surrounding the Xikrin territory. Nickel (Ni) is the primary target of extraction in the region and a potential environmental contaminant. However, Ni ores frequently contain other elements that may be released during extraction or processing, including As, Cd, Co, Cu, and Pb. Mercury (Hg), although not a primary component of Ni ores, can also be mobilized through soil disturbance, ore processing, and hydrological changes associated with mining operations, contributing to environmental dispersion. Additionally, naturally occurring elements such as Fe and Mn may appear at elevated concentrations in mining effluents. The presence of these elements among those exceeding reference values reinforces the plausibility of mining-related environmental contamination affecting the studied children.

Traditional strategies for the remediation of water contamination by toxic and potentially toxic elements typically rely on chemical treatments that have limited efficacy. While such methods may partially mitigate the effects of toxic elements, they also introduce new pollutants into the ecosystem. In light of this limitation, biological approaches have gained prominence as more sustainable and environmentally safe alternatives, employing plants and microorganisms capable of bioaccumulation and bioremediation [23]. However, this reality remains far removed for the Xikrin do Cateté people, whose environmental exposure is the result of over three decades of mining activities carried out in repeated violation of legal regulations. The violation of the Xikrin’s territorial and human rights predates even the official demarcation of their lands, which occurred only in 1991. Before this formal recognition, an area of approximately 13,000 hectares, encompassing parts of the Cateté and Itacaiúnas rivers, essential for hunting, fishing, and cultural practices, was deliberately excluded from the demarcation. Paradoxically, this same area has continued to be exploited for mining activities, primarily by private companies, without proper consultation or compensation to the Indigenous community [13].

Moulatlet et al. report that the enrichment of Cd, chromium (Cr), Cu, Fe, Hg, Mn, Ni, Pb, and zinc (Zn) may be associated with remobilization resulting from mining activities [4]. The concentrations of certain metals in sediments and water samples collected near mining areas were found to be up to 100 times higher than background environmental baseline, defined as levels without direct anthropogenic interference [4]. Although Amazonian sediments naturally contain high concentrations of lithogenic metals, the enrichment levels observed in sediments and water indicate anthropogenic remobilization and fluvial discharge from mining activities.

One of the metals showing high enrichment in mining sites was Hg. Its presence is likely due to its historical use in the amalgamation process to concentrate and extract silver (Ag) and Au from low-grade ores, a process that often results in uncontrolled environmental release [4]. Metals such as Fe and Mn were also reported at concentrations exceeding background levels by 100-fold or more in mining regions. The average concentrations of Cd, Cr, Cu, Ni, Pb, and Zn were also found to surpass average background environmental levels. This may be linked to cassiterite extraction, which mobilizes metals from rocks and soils, as well as to Au mining. Cadmium (Cd), in particular, is strongly associated with mining operations and is predominantly found in its bioavailable sedimentary fraction of anthropogenic origin [4].

In response to this alarming situation, the Federal Public Prosecutor’s Office filed a Public Civil Action against the mining company Vale S.A., as well as against the Federal Government and the State of Pará, Brazil. The lawsuit, estimated at R$ 10,000,000.00, seeks to hold the defendants accountable for the damage caused to the health and dignity of the Xikrin people. Among the requested measures are the obligation for the mining company to cover the medical treatment of affected Indigenous individuals, and the implementation of a continuous epidemiological surveillance system to monitor the health of the community. The Federal Government and the State of Pará, Brazil, have been assigned responsibility for adequately supervising mining operations and ensuring the presence of multidisciplinary teams through the Special Secretariat for Indigenous Health (SESAI of the Portuguese *Secretaria Especial de Saúde Indígena*) [13].

Although judicial measures may represent progress in terms of accountability and damage mitigation, it is essential to understand that environmental impacts transcend the legal sphere. For the Xikrin, the Cateté and Itacaiúnas rivers are not merely sources of subsistence: they are sacred spaces, essential for millennia-old rituals and for the cultural and spiritual continuity of their people. The relationship these communities have with nature goes far beyond utility; it is an ancestral, symbiotic, and profoundly spiritual connection that cannot be restored solely through financial compensation or technical programs.

Additionally, it is essential to consider the recent impact of the coronavirus disease 2019 (COVID-19) pandemic on the Indigenous population in Brazil [24–31]. Although the effects widely observed across the general population, such as increased economic, social, and health vulnerability, were significant, they were even more pronounced among Indigenous peoples. This is largely due to the fact that these communities were already facing long-standing structural challenges prior to the pandemic, including limited access to healthcare services, deforestation, land encroachment, and environmental pollution, as well as marked ethno-racial inequalities [28].

Importantly, the reference values adopted in this study correspond to those used by the certified analytical laboratory responsible for the biomonitoring analyses. According to the laboratory’s technical documentation, these cutoffs were informed by WHO guidance and international toxicological literature, and are formally documented in the public records of the Civil Public Action filed by the Federal Public Prosecutor’s Office. As these values are legally embedded in the ongoing judicial process and define the operational thresholds applied to the reported results, the numerical reference values could not be modified.

It is acknowledged that the WHO does not provide specific guideline values for hair concentrations for many elements, and this limitation is now explicitly stated. World Health Organization (WHO) documents were used solely as toxicological and public-health frameworks to contextualize known health effects, toxicological relevance, and the absence or presence of recognized safe exposure thresholds.

Hair was employed as a biomarker of internal exposure and bioaccumulation, and no direct quantitative equivalence was assumed between environmental and biological matrices. Accordingly, processes such as bioaccumulation and biomagnification preclude direct matrix-to-matrix comparisons. To enhance transparency, peer-reviewed literature reporting reference ranges and interpretative frameworks for elemental analysis in hair is cited to support the adopted interpretative approach.

## Limitations

From a methodological perspective, an important limitation is that the data are presented in aggregate form, as individual-level information for all analyzed children and adults is not publicly available, which limits detailed exposure–risk assessments stratified by age or other demographic variables. Additionally, there are no universally accepted reference ranges for toxic and potentially toxic elements in human hair, and reported concentrations may vary according to digestion protocols, population characteristics, and analytical techniques. Importantly, the reference values adopted in the present study were defined based on information obtained through direct contact with the laboratory responsible for the analyses, as these values were those used by the company in the interpretation of the reported results; the same reference values were also explicitly reported in the public documentation associated with Civil Public Action n° 1,001,462 − 67.2025.4.01.3901, currently under review by the 1st Federal Civil and Criminal Court of the Judicial Subsection of Marabá, State of Pará, Brazil.

The study design captures exposure at a single time point, preventing a comprehensive assessment of chronic exposure, temporal variability, or seasonal fluctuations. Potential confounding factors, such as sex, age, dietary habits, occupational activities, and other environmental sources of contamination, could not be fully controlled. Although total concentrations of toxic and potentially toxic elements were measured, information on bioavailability, chemical speciation, and long-term biological retention is lacking. Environmental sampling was limited, as parallel measurements in soil, water, or sediments were not systematically available, restricting direct correlations between environmental contamination and human exposure.

While a substantial proportion of the Xikrin population was included, other Indigenous or riparian communities potentially affected by mining activities in the region were not assessed, limiting the generalizability of the findings. Although ICP-AES and GFAAS are robust analytical techniques, measurement uncertainty and potential matrix effects may influence element quantification. Ethical and privacy constraints prevented disclosure of certain individual data, further limiting granular interpretation. Moreover, socioeconomic and cultural factors, such as food insecurity, limited access to healthcare, and specific cultural practices, may modulate the health impacts of exposure but were not quantitatively assessed. Historical exposure, including prenatal and early-life periods, was inferred rather than directly measured, potentially underestimating cumulative lifetime exposure. The absence of implemented remediation strategies in the territory also limits the study’s ability to evaluate immediate mitigation outcomes.

A key methodological limitation of this study is the absence of detailed QA/QC information for the elemental analyses. The biomonitoring data were obtained from an official technical report generated by a certified commercial laboratory and incorporated into public judicial documentation, and the authors did not have access to raw analytical data or full methodological records. Consequently, information regarding the number of analytical replicates, calibration curve coefficients of determination, certified reference materials and element-specific recoveries, internal standards, spike procedures, matrix interference correction strategies, limits of detection (LOD), limits of quantification (LOQ), and the handling of results below these thresholds was not available. In addition, although GFAAS was used for Se and ICP-AES for other elements, the rationale for this distinction and details regarding collision or reaction cell use for elements such as As could not be verified. These limitations preclude a comprehensive independent assessment of analytical performance at the trace and ultra-trace level and require that the findings be interpreted as evidence of population-level exposure patterns rather than as a full analytical validation of elemental concentrations. Despite these constraints, the dataset reflects results generated by a certified laboratory and currently used in regulatory and judicial decision-making, supporting its relevance for public health interpretation while underscoring the need for future studies with fully documented QA/QC procedures.

## Strengths

This study is strengthened by its robust sample size and extensive population coverage, including biomonitoring data from 732 Indigenous individuals, representing nearly half of the entire Xikrin do Cateté population. This high level of participation provides unprecedented statistical power and enables reliable population-level inferences regarding exposure to toxic and potentially toxic elements. The analytical rigor of the investigation is further reinforced by the use of internationally recognized gold-standard techniques for toxic and potentially toxic elements quantification, namely ICP-AES and GFAAS, enhancing the accuracy, reproducibility, and scientific reliability of the findings.

A major strength of the study lies in its multidimensional integration of environmental and health data. Biological measurements were interpreted alongside extensive environmental, territorial, and legislative context, including deforestation processes, hydrological disruption, legal and illegal mining activities, and broader socio-political vulnerabilities. Rather than focusing on a single contaminant, the study characterizes complex multi-element exposure profiles, capturing the cumulative burden of contamination and demonstrating patterns consistent with the geochemical signature of Ni-focused mining activities in the region. This integrative approach strengthens the plausibility of environmentally mediated exposure pathways and substantially increases the translational relevance of the findings for public health surveillance, regulatory action, and policy decision-making.

The inclusion of vulnerable and historically under-studied populations represents another key strength. All children included in the analysis presented at least one toxic or potentially toxic element above reference values, underscoring exposure among the most sensitive population group and reinforcing concerns regarding prenatal and early-life exposure. Interpretations were aligned with internationally recognized public health frameworks, ensuring global comparability, adherence to established toxicological standards, and strengthening the credibility of the results in regulatory, judicial, and public health contexts.

An additional strength is the use of a publicly accessible and legally recognized data source, as all analytical results were derived from documents filed in an official Public Civil Action. This guarantees data transparency, legal robustness, and enables independent verification by scientific, judicial, and public health institutions. Collectively, the comprehensive exposure data, combined with the documented clinical risk profiles, fill a critical gap in the literature by providing one of the most extensive datasets on toxic or potentially toxic elements exposure among Indigenous peoples in the Brazilian Amazon. These findings offer compelling scientific evidence supporting the classification of the situation in the Xikrin do Cateté Indigenous Territory as a public health emergency requiring immediate, coordinated, and intersectoral intervention.

## Conclusions

This study provides compelling evidence that anthropogenic mining activities in the Xikrin do Cateté Indigenous Territory have resulted in widespread environmental contamination and pervasive human exposure to toxic and potentially toxic elements. The combination of biological, environmental, and socio-cultural data demonstrates that these communities are experiencing a public health emergency, with multi-generational implications for health, nutrition, and cultural continuity.

From a scientific standpoint, the data confirm that contamination is not isolated, but systemic, involving multiple elements with neurotoxic, nephrotoxic, and hematological effects, detectable even in the youngest members of the population. The study highlights the importance of integrating biomonitoring, environmental assessment, and epidemiological surveillance to accurately characterize exposure pathways and health risks.

From a social and ethical perspective, the findings emphasize that the impact of mining extends beyond measurable toxicity. The contamination disrupts traditional food systems, compromises cultural practices, and violates Indigenous rights to land, water, and environmental sovereignty. Remediation efforts cannot be limited to chemical or technological interventions; they must incorporate community engagement, legal accountability, and culturally sensitive restoration strategies.

Collectively, this work underscores the urgent need for intersectoral policy action, combining environmental regulation, public health surveillance, and Indigenous governance. The Xikrin case exemplifies the broader challenges facing Indigenous and riparian populations worldwide, revealing how extractive industries, if inadequately monitored and regulated, threaten both ecological integrity and human rights. Addressing these challenges requires not only scientific evidence but also political will and ethical commitment to protect vulnerable populations and sustain the planet’s biodiversity and cultural heritage.

As Chief Kayapó Raoni Metuktire, one of Brazil’s most respected Indigenous leaders, once said:

*We all breathe the same air*,* we all drink the same water*,* we all live on the same Earth. We must protect it*.

## Supplementary Information

Supplementary Material 1.

## Data Availability

The data supporting the findings of this study are publicly available through the records of the Federal Court of Brazil. Specifically, the data are part of the public documentation associated with Civil Public Action n° 1,001,462 − 67.2025.4.01.3901, currently under review by the 1st Federal Civil and Criminal Court of the Judicial Subsection of Marabá, Pará (*1ª Vara Federal Cível e Criminal da Seção Judiciária de Marabá*,* Pará*), Brazil. All relevant legal and environmental information used in the research can be accessed through the official Brazilian Federal Justice portal (*Justiça Federal*) or upon reasonable request to the court’s public records office. However, access to raw analytical data, detailed laboratory protocols, and quality assurance/quality control procedures is restricted, as these materials are embedded within judicial and technical reports submitted under legal confidentiality and procedural constraints. Consequently, granular methodological details and primary laboratory datasets cannot be independently retrieved or reproduced outside the scope of the ongoing legal proceedings.
