# Supplementary material for: Mining Activities Have Exposed Individuals of an Indigenous Tribe in Brazil to Toxic and Potentially Toxic Elements
Source: J Racial Ethn Health Disparities. 2026 Mar 16;13(3):1611–27. doi: 10.1007/s40615-026-02918-y (PMC13157454; doi:10.1007/s40615-026-02918-y)
Supplement: Supplementary file 1 — Supplementary Material 1. [file 40615_2026_2918_MOESM1_ESM.docx]

Journal of Racial and Ethnic Health Disparities

COMMENTS FOR THE AUTHOR:

**Reviewer #1:** The authors have addressed the issues outlined in my review.

**Reply:** We sincerely thank the reviewer for the careful evaluation of our manuscript and for acknowledging that the concerns raised in the previous review have been adequately addressed. We appreciate the constructive feedback and valuable support, which significantly contributed to improving the quality and clarity of the manuscript.

**Reviewer 3**

Dear Editor and Reviewers,

We would like to begin by sincerely thanking you for the careful evaluation of our manuscript and for the depth and rigor of the comments provided. We also respectfully acknowledge the methodological and analytical limitations identified. We apologize for the constraints present in the dataset and documentation available to us, which inevitably restricted the level of analytical and toxicological detail that could be presented in several sections of the manuscript.

These limitations are intrinsic to the nature of the data source. The study is based entirely on secondary information derived from official technical and judicial documentation, without access to individual-level records or full laboratory QA/QC reports. Precisely because of these constraints, we deliberately chose the Perspective article format and adopted a cautious, conservative, and transparent interpretative approach throughout the manuscript. We substantially expanded and clarified the Limitations section and revised the text in multiple areas to avoid overinterpretation and to clearly separate documented findings from interpretative context.

At the same time, we respectfully emphasize the public health and ethical relevance of disseminating these findings despite the acknowledged limitations. The data concern a highly vulnerable Indigenous population exposed to complex environmental pressures in a mining-impacted territory. Even with methodological restrictions, the scale of the biomonitoring dataset and the consistency of the population-level exposure signals provide important grounds for scientific discussion, risk awareness, and the stimulation of more detailed and methodologically robust future investigations. In this sense, the proposed study model, integrative, document-based, and perspective-oriented, was intentionally selected as the most responsible and transparent way to present the available evidence without exceeding what the data can support.

We are grateful for the opportunity to revise the manuscript and believe that the changes made in response to the reviewers’ comments have significantly improved its clarity, accuracy, transparency, and scientific balance.

Sincerely,

The Authors

**Reviewer #3:** This paper is interesting, but has several major flaws, including a lack of understanding on metal toxicology. There are several things that MUST be clarified in order for this paper to achieve a minimum analytical quality and, consequently, adequate conclusions.

**Reply:** We sincerely thank the reviewer for the careful reading of our manuscript and for the critical comments provided. We fully acknowledge that the study presents important limitations, and we appreciate the opportunity to clarify these aspects and to improve the manuscript accordingly.

This investigation is entirely based on secondary data obtained from public documentation associated with Civil Public Action No. 1001462-67.2025.4.01.3901, which is currently under review by the 1st Federal Civil and Criminal Court of the Judicial Subsection of Marabá, Pará, Brazil. As detailed throughout the manuscript, individual-level data for each Indigenous participant were not made available, and the laboratory data, particularly the analytical methods and protocols, were not fully described in the official documentation.

In an effort to mitigate these constraints, we contacted the laboratory responsible for the analyses and were able to obtain only minimal methodological information, which has now been explicitly described in the revised text. Given these inherent data limitations, we deliberately adopted a cautious and conservative interpretation of the findings and substantially expanded and strengthened the limitations section to transparently reflect these issues.

We agree with the reviewer that the lack of detailed toxicological and analytical information restricts the depth of mechanistic interpretation. However, we believe that, despite these constraints, the urgent dissemination of these data is scientifically and ethically justified. The population under study represents a highly vulnerable Indigenous community that has been exposed to multiple social and environmental stressors linked to external factors such as mining. Presenting these findings, even with acknowledged limitations, may provide critical support for public health surveillance, policy discussion, and future, more robust investigations focused on this population.

We are grateful for the reviewer’s comments, which have helped us refine the manuscript and clarify its scope, limitations, and intended contribution.

- Please indicate if you used Chat GPT or similar in a separate section at the end of the manuscript, as per journal guidelines. If so, indicate what you used it for, and confirm in that section you have critically revised all statements given by this AI and based the statements on scientific literature. Many instances are very clearly "Chat GPT-style", such as underscore, elevated, the use of hyphens, etc. Also, alter your "Limitations" and "Strengths" sections to running text, not bullet points. (Correct the word, it is written as Strenghts).

**Reply:** We thank the reviewer for raising this important point and for the careful attention to transparency regarding the use of artificial intelligence tools.

We would like to clarify that no generative artificial intelligence tools (such as ChatGPT or similar platforms) were used to create, modify, or generate scientific content, arguments, interpretations, or conclusions in this manuscript. The only digital support tool employed during the revision process was Grammarly, integrated into Microsoft Word, which was used exclusively for grammatical corrections, spelling, punctuation, and language fluency, without altering the scientific meaning or introducing new content. All revisions were manually reviewed and approved by the authors.

To comply with the journal’s guidelines and ensure full transparency, we propose the inclusion of the following statement in a separate section at the end of the declarations (Title Page):

***Use of artificial intelligence tools:*** *The authors declare that no generative artificial intelligence tools (such as ChatGPT or similar platforms) were used in the writing, analysis, or interpretation of the scientific content of this manuscript. Grammarly^®^, integrated into Microsoft Word, was used solely to assist with grammatical and linguistic corrections. All revisions were critically reviewed by the authors, and the scientific content remains entirely based on the authors’ original work and on the cited scientific literature. NotebookLM (Google LLC) was used under an institutional license provided by the University of São Francisco (USF of the Portuguese Universidade São Francisco) through Google Workspace for Education. This license authorizes faculty and students to use the tool for academic and research purposes. The platform supported the organization and analysis of academic materials, and one figure included in this work was generated using the application under this licensed access. Data usage followed institutional policies for privacy, security, and responsible use of digital tools.*

Regarding the reviewer’s observation about stylistic patterns that may resemble AI-generated text (e.g., hyphenation or word choice), we would like to emphasize that these elements reflect standard academic English usage and editorial refinement during language revision, rather than automated text generation. Nonetheless, we carefully reviewed the manuscript again to ensure consistency in writing style and clarity.

In addition, as suggested, we have revised the “Limitations” and “Strengths” sections, converting them from bullet points into continuous running text to better align with the journal’s formatting and stylistic requirements. We have also corrected the typographical error in the section title, replacing *“Strenghts”* with *“Strengths”*.

As indicated, we will next provide the revised versions of the Limitations and Strengths sections for further review and refinement.

We appreciate the reviewer’s constructive feedback, which has contributed to improving the transparency, clarity, and overall quality of the manuscript.

***4. Limitations***

*From a methodological perspective, an important limitation is that the data are presented in aggregate form, as individual-level information for all analyzed children and adults is not publicly available, which limits detailed exposure–risk assessments stratified by age or other demographic variables. Additionally, there are no universally accepted reference ranges for toxic and potentially toxic elements in human hair, and reported concentrations may vary according to digestion protocols, population characteristics, and analytical techniques. Importantly, the reference values adopted in the present study were defined based on information obtained through direct contact with the laboratory responsible for the analyses, as these values were those used by the company in the interpretation of the reported results; the same reference values were also explicitly reported in the public documentation associated with Civil Public Action n° 1001462-67.2025.4.01.3901, currently under review by the 1st Federal Civil and Criminal Court of the Judicial Subsection of Marabá, State of Pará, Brazil.*

*The study design captures exposure at a single time point, preventing a comprehensive assessment of chronic exposure, temporal variability, or seasonal fluctuations. Potential confounding factors, such as sex, age, dietary habits, occupational activities, and other environmental sources of contamination, could not be fully controlled. Although total concentrations of toxic and potentially toxic elements were measured, information on bioavailability, chemical speciation, and long-term biological retention is lacking. Environmental sampling was limited, as parallel measurements in soil, water, or sediments were not systematically available, restricting direct correlations between environmental contamination and human exposure.*

*While a substantial proportion of the Xikrin population was included, other Indigenous or riparian communities potentially affected by mining activities in the region were not assessed, limiting the generalizability of the findings. Although ICP-AES and GFAAS are robust analytical techniques, measurement uncertainty and potential matrix effects may influence element quantification. Ethical and privacy constraints prevented disclosure of certain individual data, further limiting granular interpretation. Moreover, socioeconomic and cultural factors, such as food insecurity, limited access to healthcare, and specific cultural practices, may modulate the health impacts of exposure but were not quantitatively assessed. Historical exposure, including prenatal and early-life periods, was inferred rather than directly measured, potentially underestimating cumulative lifetime exposure. The absence of implemented remediation strategies in the territory also limits the study’s ability to evaluate immediate mitigation outcomes.*

*A key methodological limitation of this study is the absence of detailed QA/QC information for the elemental analyses. The biomonitoring data were obtained from an official technical report generated by a certified commercial laboratory and incorporated into public judicial documentation, and the authors did not have access to raw analytical data or full methodological records. Consequently, information regarding the number of analytical replicates, calibration curve coefficients of determination, certified reference materials and element-specific recoveries, internal standards, spike procedures, matrix interference correction strategies, limits of detection (LOD), limits of quantification (LOQ), and the handling of results below these thresholds was not available. In addition, although GFAAS was used for Se and ICP-AES for other elements, the rationale for this distinction and details regarding collision or reaction cell use for elements such as As could not be verified. These limitations preclude a comprehensive independent assessment of analytical performance at the trace and ultra-trace level and require that the findings be interpreted as evidence of population-level exposure patterns rather than as a full analytical validation of elemental concentrations. Despite these constraints, the dataset reflects results generated by a certified laboratory and currently used in regulatory and judicial decision-making, supporting its relevance for public health interpretation while underscoring the need for future studies with fully documented QA/QC procedures.*

***5. Strengths***

*This study is strengthened by its robust sample size and extensive population coverage, including biomonitoring data from 732 Indigenous individuals, representing nearly half of the entire Xikrin do Cateté population. This high level of participation provides unprecedented statistical power and enables reliable population-level inferences regarding exposure to toxic and potentially toxic elements. The analytical rigor of the investigation is further reinforced by the use of internationally recognized gold-standard techniques for toxic and potentially toxic elements quantification, namely ICP-AES and GFAAS, enhancing the accuracy, reproducibility, and scientific reliability of the findings.*

*A major strength of the study lies in its multidimensional integration of environmental and health data. Biological measurements were interpreted alongside extensive environmental, territorial, and legislative context, including deforestation processes, hydrological disruption, legal and illegal mining activities, and broader socio-political vulnerabilities. Rather than focusing on a single contaminant, the study characterizes complex multi-element exposure profiles, capturing the cumulative burden of contamination and demonstrating patterns consistent with the geochemical signature of Ni-focused mining activities in the region. This integrative approach strengthens the plausibility of environmentally mediated exposure pathways and substantially increases the translational relevance of the findings for public health surveillance, regulatory action, and policy decision-making.*

*The inclusion of vulnerable and historically under-studied populations represents another key strength. All children included in the analysis presented at least one toxic or potentially toxic element above reference values, underscoring exposure among the most sensitive population group and reinforcing concerns regarding prenatal and early-life exposure. Interpretations were aligned with internationally recognized public health frameworks, ensuring global comparability, adherence to established toxicological standards, and strengthening the credibility of the results in regulatory, judicial, and public health contexts.*

*An additional strength is the use of a publicly accessible and legally recognized data source, as all analytical results were derived from documents filed in an official Public Civil Action. This guarantees data transparency, legal robustness, and enables independent verification by scientific, judicial, and public health institutions. Collectively, the comprehensive exposure data, combined with the documented clinical risk profiles, fill a critical gap in the literature by providing one of the most extensive datasets on toxic or potentially toxic elements exposure among Indigenous peoples in the Brazilian Amazon. These findings offer compelling scientific evidence supporting the classification of the situation in the Xikrin do Cateté Indigenous Territory as a public health emergency requiring immediate, coordinated, and intersectoral intervention.*

- Do not begin paragraphs with abbreviations (Ex: Ni is the primary target of extraction… alter to Nickel…).

**Reply:** Thank you for this observation. The correction has been made, and all paragraphs have been revised to avoid beginning with abbreviations. Chemical element names are now written in full at the beginning of sentences, with abbreviations introduced subsequently when appropriate.

- You don't have any subsections, only "TEXT" at the beginning. Correct.

**Reply:** Thank you for this comment. The manuscript was intentionally submitted as a Perspective article due to the inherent limitations related to the nature of the data source, including the use of secondary data, the absence of individual-level information, and the limited availability of detailed methodological descriptions regarding laboratory analyses and reference cut-off values. In this article format, a continuous narrative without conventional subsections is commonly adopted.

Nevertheless, in order to better align with the reviewer’s suggestion and to improve clarity and readability, we have revised the manuscript and introduced thematic subsections to organize the text. We sincerely appreciate this recommendation, as the revised structure has improved the overall flow and accessibility of the manuscript.

*1. Mining, Toxic and Potentially Toxic Elements, and Environmental Contamination in the Amazon*

*2. The Xikrin do Cateté Territory: Environmental Pressure, Mining Activities, and Socio-Cultural Impacts*

*3. Biomonitoring Evidence: Analytical Methods, Exposure Profiles, and Public Health Implications*

*4. Limitations*

*5. Strengths*

*6. Conclusion*

*7. Reference*

- Line 110 - "Human exposure to potentially toxic elements can occur via multiple routes, including direct contact with contaminated wate" - And to essential elements in excess and TOXIC ONLY as well, alter.

**Reply:** We thank the reviewer for this important clarification. The sentence has been revised to explicitly distinguish between potentially toxic elements and essential elements that may become toxic only when present in excess. The text now clearly states that human exposure occurs through multiple environmental and dietary routes and emphasizes that elements such as iron, zinc, copper, manganese, nickel, and cobalt are physiologically essential but can exert toxic effects at elevated concentrations. This revision improves conceptual accuracy and aligns the text with established toxicological frameworks.

*Human exposure to toxic and potentially toxic elements can occur via multiple routes, including direct contact with contaminated water, air, or soil, as well as through the consumption of contaminated food, such as fish and vegetables grown in affected areas. In addition, essential elements that are fundamental to biological processes, such as iron (Fe), zinc (Zn), copper (Cu), manganese (Mn), nickel (Ni), and cobalt (Co), may become toxic when present in excess. The bioaccumulation of these metals in the human body can lead to significant levels of toxicity, with clinical manifestations depending on the chemical element, concentration, and duration of exposure (****Table 1****). Even at subclinical levels, chronic exposure poses health risks. The main organs and systems affected include the central nervous system, kidneys, liver, immune system, and cardiovascular system [9].*

Much more details on the QA/QC of the metal analysis are required, I simply can't comment on the Quality Control/Quality Assurance of the method with no data! You have SEVERAL elements that suffer matrix interferences or that are present at ppt levels, this is important. Insert at least the following:
- Number of replicates

- Equipment conditions

- What certified reference materials or spikes were used

- What were the curve coefficients of determination

- What were the certified reference material used and their recoveries for each element
- What were the LOD and LOQ for each element and how were calculated. All elements cannot have the same LOD and/or LOQ.

- What was the internal standard and how much was used? If no spikes and/or CRM were used in the metal analyses, the paper cannot be published.  P.S.

- CRM are not standards used to prepare calibration curves.

- GFAAS reference value was applied only for selenium (Se) - Why? Did the As determinations use a collision cell? This information is paramount.

- Also, how many samples were below the LOQ? Or the LOD? Were they used in the statistical analyses/graphs?

Reply: We thank the reviewer for this rigorous and technically important assessment. We fully acknowledge that the manuscript does not contain detailed Quality Assurance/Quality Control (QA/QC) information at the analytical level requested, and we agree that such details are fundamental for a complete evaluation of trace and ultra-trace elemental analyses.

The present study is based exclusively on secondary data obtained from an official technical report generated by a certified commercial laboratory (BioMinerais, Campinas, São Paulo, Brazil), which was commissioned by public authorities and later incorporated into the documentation of an ongoing Civil Public Action. As such, the authors did not have access to the full analytical raw data, instrument logs, or internal QA/QC documentation, including the number of analytical replicates, calibration curve coefficients of determination, certified reference material (CRM) recoveries by element, limits of detection (LOD) and quantification (LOQ), internal standards, spike recoveries, or collision/reaction cell configurations.

According to the technical report, elemental quantification was performed using ICP-AES for most elements and GFAAS specifically for selenium (Se), following the laboratory’s internal protocols. However, no further methodological justification was provided regarding the exclusive use of GFAAS for Se, nor were details available on whether collision cell technology was applied for elements such as arsenic. Likewise, information on matrix interference correction, element-specific LOD/LOQ values, or the treatment of results below these thresholds was not disclosed in the public documentation.

Because these analyses were conducted independently by the laboratory prior to the authors’ involvement, and because the dataset is legally constrained and publicly available only in aggregated and summarized form, it was not possible to retrospectively obtain or reconstruct this level of analytical detail. Importantly, the authors did not perform the chemical analyses themselves and therefore cannot introduce methodological specifications that are not explicitly documented in the source material.

In response to the reviewer’s concern, we have now explicitly clarified these constraints in the manuscript and substantially strengthened the Limitations section to transparently state the absence of detailed QA/QC information and its implications for the interpretation of the results. We emphasize that the study’s objective is not to validate or optimize an analytical method, but to interpret a large-scale biomonitoring dataset already used in regulatory and judicial contexts to highlight public health implications for a highly vulnerable population.

*A key methodological limitation of this study is the absence of detailed QA/QC information for the elemental analyses. The biomonitoring data were obtained from an official technical report generated by a certified commercial laboratory and incorporated into public judicial documentation, and the authors did not have access to raw analytical data or full methodological records. Consequently, information regarding the number of analytical replicates, calibration curve coefficients of determination, certified reference materials and element-specific recoveries, internal standards, spike procedures, matrix interference correction strategies, limits of detection (LOD), limits of quantification (LOQ), and the handling of results below these thresholds was not available. In addition, although GFAAS was used for Se and ICP-AES for other elements, the rationale for this distinction and details regarding collision or reaction cell use for elements such as As could not be verified. These limitations preclude a comprehensive independent assessment of analytical performance at the trace and ultra-trace level and require that the findings be interpreted as evidence of population-level exposure patterns rather than as a full analytical validation of elemental concentrations. Despite these constraints, the dataset reflects results generated by a certified laboratory and currently used in regulatory and judicial decision-making, supporting its relevance for public health interpretation while underscoring the need for future studies with fully documented QA/QC procedures.*

Table 3- What are the bold values? In fact, why on earth are you depicting values for TWO people? The data should be medians/averages stratified by age and sex! Outliers could be commented on, but simply two individuals indicates absolutely nothing. You state " These children were not chosen because their findings were isolated or atypical, but rather because they represented the pattern of multi-elemental contamination described across the broader population, while also illustrating the magnitude of the contamination among the youngest individuals." - Where do you state their pattern is similar across the broader population? What is that pattern? Children excrete elements differentially from adults! Sex is also important.

**Reply:** We thank the reviewer for this detailed and important comment, which allowed us to further clarify the rationale and presentation of Table 3.

The bold values in Table 3 indicate concentrations that exceed the reference values adopted for interpretation. These reference values were those used by the analytical laboratory and explicitly reported both through direct communication with the laboratory and in the public documentation associated with Civil Public Action n° 1001462-67.2025.4.01.3901, as described in the Methods and Limitations sections.

We fully agree that, from an epidemiological and analytical standpoint, population-level summaries such as medians or means stratified by age and sex would be preferable. However, this was not possible in the present study, as the publicly available data are reported exclusively in aggregate form, without access to individual-level values stratified by age, sex, or other demographic variables. This fundamental limitation is now explicitly emphasized throughout the manuscript and was one of the main reasons the work was submitted as a Perspective article rather than an original analytical study.

The inclusion of data from two children in Table 3 does not aim to provide statistical inference or represent outliers. Rather, these cases were the only individual-level examples made publicly available in the official technical report, and their presentation serves an illustrative and contextual purpose, consistent with the perspective format. Importantly, these children were highlighted in the original report because they exemplify the multi-element exposure profile observed at the population level, characterized by the concurrent elevation of several potentially toxic elements rather than isolated single-metal exposure. This pattern is documented in the aggregate results, which show that 98.5% of the analyzed population presented at least one element above reference values and that multiple elements were simultaneously elevated across the cohort.

We acknowledge the reviewer’s correct observation that children differ from adults in toxicokinetics and element excretion, and that sex-related differences are also relevant. Unfortunately, the lack of disaggregated individual data precluded any stratified analysis by age or sex. This constraint is now more clearly stated in the Limitations section, and we have intentionally avoided extrapolating mechanistic or causal conclusions from these two illustrative cases.

To avoid misinterpretation, we have revised the text to reinforce that Table 3 is illustrative, not inferential, and that the conclusions of the manuscript are based on population-level aggregate findings, not on these two individual cases. We believe that, within the context of a perspective article grounded in publicly available legal documentation, this approach balances transparency, ethical constraints, and scientific responsibility.

*The inclusion of data from two children in* ***Table 3*** *does not aim to provide statistical inference or represent outliers. Rather, these cases were the only individual-level examples made publicly available in the official technical report, and their presentation serves an illustrative and contextual purpose. Importantly, these children were highlighted in the original report because they exemplify the multi-element exposure profile observed at the population level, characterized by the concurrent elevation of several toxic and potentially toxic elements rather than isolated single-metal exposure. This pattern is documented in the aggregate results, which show that 98.5% of the analyzed population presented at least one element above reference values and that multiple elements were simultaneously elevated across the cohort.*

*It is important to acknowledge that children differ substantially from adults in toxicokinetics, including absorption, distribution, metabolism, and excretion of chemical elements, and that sex-related biological differences may further influence exposure profiles and biological responses. These factors are particularly relevant in the context of toxic and potentially toxic elements, for which age- and sex-specific variability may affect internal dose and health effects. However, the present study was limited by the exclusive availability of aggregated data derived from public documentation, which precluded stratified analyses according to age, sex, or other individual-level characteristics. Consequently, no age- or sex-specific inferences could be performed. In light of these constraints, the individual cases presented are intended solely for illustrative purposes, and no mechanistic or causal conclusions were extrapolated from these examples.*

- Remove all instances of "Heavy metal", this is incorrect according to IUPAC standards. Also remove all instances of "Trace elements", alter to metals as several are not trace. (Table 2 and several figures). You yourself state this is inadequate but use the term in figures, tables, and in the text. Also, arsenic and selenium are not metals, but metalloids/semimetals/ametals, alter.

**Reply:** We thank the reviewer for this important observation. In accordance with IUPAC recommendations, all instances of the terms “heavy metal” and “trace elements” have been removed throughout the manuscript. We have consistently adopted the term “potentially toxic elements”, which more accurately reflects the chemical and toxicological diversity of the analyzed elements. In addition, arsenic and selenium were correctly classified as metalloids across the text, tables, and figures.

- "Hair samples were collected and analyzed through hair mineralogram (mineralography), a method that enables the identification and quantification of toxic elements accumulated in the human body." - And essential elements in excess. Simply state: "obtain elemental profiles" or similar.

**Reply:** We thank the reviewer for this important suggestion. The sentence has been revised to avoid restricting the method to the identification of toxic elements only. It now describes hair mineralogram analysis more appropriately as a technique to obtain elemental profiles in biological samples, including potentially toxic elements and essential elements in excess. This change improves the technical accuracy and neutrality of the description.

*Hair samples were collected and analyzed using hair mineralogram (mineralography), allowing the assessment of elemental profiles, including both potentially toxic elements and essential elements in excess.*

- Correct units to mg kg-1, ug kg-1, etc according to IUPAC standards. Remove all instances of a bar between units, including in the figures and tables, and kg should NOT be capitalized. (Check all tables and figures).

Reply: We thank the reviewer for this important comment. All units throughout the manuscript, including the main text, tables, and figures, have been carefully revised to comply with IUPAC and SI standards. Units have been standardized to the recommended format using negative exponents (e.g., mg kg⁻¹, µg kg⁻¹), all instances of slashes or bars between units have been removed, and “kg” has been consistently written in lowercase. These corrections were applied uniformly across all tables and figures to ensure full consistency and adherence to international standards.

- Your map should indicate the continent, country, and then zoom in on the region. A north is also required, and coordinates would be good.

**Reply:** Thank you for the guidance. The requested corrections have been made. The map now indicates the continent and country and includes a zoomed view of the study region. A north arrow and geographic coordinates have also been added.

- Graphical abstract - You cannot state that "mining activities have exposed ALL members of the tribe to potentially toxic elements", this is simply NOT true. Several elements may originate from MANY other sources than mining (Hg for example is known as typically high in Amazon soils, so although mining may increase levels, several exposure routes are possible). Soften this statement.

**Reply:** We thank the reviewer for this important and well-taken observation. We fully agree that stating that mining activities have exposed *all* members of the Indigenous community is overly assertive and does not adequately reflect the complexity of exposure pathways in the Amazon region. As correctly highlighted, several potentially toxic elements may originate from multiple natural and anthropogenic sources, including geogenic background levels and other environmental processes, and mining represents one, but not the sole, contributor to exposure.
To address this concern, we have softened and revised the statement in the graphical abstract to avoid causal overstatement and to better reflect the multifactorial nature of exposure. The revised wording emphasizes the association between environmental contamination in mining-impacted territories and widespread exposure, without implying exclusivity or universality.

*Evidence of Environmental Contamination and Population-Level Exposure to Toxic and Potentially Toxic Elements in an Indigenous Territory Affected by Mining Activities in Brazil*

- What document has all the reference guidelines from the WHO? I believe this is not true. You did not REFERENCE this, only stated it's according to the lab that conducted your analyses. This is not the correct way to reference this. Also, check, I believe that there are not so many established references by that agency for so many elements. I'd like to check.  BUT - "Although specific guideline values are not available for every element analyzed, the WHO Guidelines for Drinking-water Quality and the associated Chemical Risk Assessment documents constitute the most widely recognized international benchmarks for evaluating human exposure to metals and metalloids in environmental and biological matrices." - You used WATER VALUES to compare HAIR values??? This is very very strange and to me invalidates the entire text if done. You can't state that a value in water, if  in hair, if over a value in water, indicates exposure, you aren't accounting for bioaccumulation, biomagnification, biodilution processes. Please explain your rationale.

**Reply:** We thank the reviewer for this careful and important evaluation. We acknowledge that the initial wording may have led to confusion regarding the origin and application of the reference values, and we apologize for any misunderstanding this may have caused.

The reference values used in the present study were those explicitly adopted by the certified analytical laboratory responsible for the biomonitoring analyses, which reported that these cutoffs were informed by World Health Organization (WHO) guidance and international toxicological literature. These same reference values are formally documented in the public records of the Civil Public Action filed by the Federal Public Prosecutor’s Office and are currently being used as operational cutoffs for judicial and regulatory decision-making. For this reason, we are not able to modify the numerical reference values themselves, as they are legally embedded in the ongoing public action and define the thresholds applied to the reported results.

We fully agree with the reviewer that the WHO does not provide specific guideline values for hair concentrations for many elements, and we have revised the text to explicitly state this limitation. Importantly, we clarify that water guideline values were not directly applied to hair concentrations, nor were hair values interpreted as exceedances of drinking-water standards. Instead, WHO documents (including the *Guidelines for Drinking-water Quality* and Chemical Risk Assessment reports) were cited exclusively as toxicological and public-health frameworks to contextualize relative toxicological relevance, known health effects, and the absence or presence of recognized safe exposure thresholds for specific elements.

To address the reviewer’s concern, we have substantially revised the Methods, Results interpretation, and Limitations sections to make clear that hair is used as a biomarker of internal exposure and bioaccumulation, and that no direct quantitative equivalence was assumed between concentrations in environmental matrices (such as water) and biological matrices (hair). We now explicitly state that processes such as bioaccumulation, biomagnification, and biodilution preclude any direct matrix-to-matrix comparison.

Additionally, we have strengthened the manuscript by incorporating peer-reviewed literature that reports reference ranges, background values, and interpretative frameworks for hair elemental analysis. These references are now cited directly in the text to ensure transparency and allow readers to independently verify the scientific basis for the adopted interpretation.

We appreciate the reviewer’s comments, which significantly improved the clarity, rigor, and transparency of the manuscript, particularly regarding the distinction between legal-operational reference values and toxicological interpretation.

- After defining an acronymn, you may use the acronymn throughout the text. You did not do so for example, for arsenic (line 94). Either define the acronymn here if it's its first appearance, or use it here if it was defined before.

**Reply:** We thank the reviewer for pointing this out. The acronym usage has been carefully revised throughout the manuscript to ensure consistency. For arsenic, the chemical symbol (As) is now defined at its first occurrence and subsequently used consistently in the text. All other elements were similarly checked to confirm that acronyms are either properly defined at first mention or written in full when appearing for the first time, in accordance with standard scientific writing conventions.

- A total of 32 chemical elements were found at elevated levels, 22 of which are metals or metalloids.  This sentence makes no sense, what is "an ELEVATED level"? You state "EXCESSIVE" is defined as over WHo limits. But you used the word ELEVATED. Also, all determined elements are either metals or metalloids, what do you mean?

**Reply:** We thank the reviewer for highlighting this important point. We agree that the term “elevated” was imprecise and could lead to conceptual ambiguity. To address this, the text has been revised to clearly state that exceedance was defined in relation to the reference values adopted in the study, rather than using a vague qualitative descriptor. In addition, we now explicitly clarify that the reference values were defined based on information obtained through direct contact with the analytical laboratory responsible for the analyses and on the reference thresholds reported in the public documentation associated with Civil Public Action n° 1001462-67.2025.4.01.3901, currently under review by the 1st Federal Civil and Criminal Court of the Judicial Subsection of Marabá, Pará.

*In the documentation associated with Civil Public Action n° 1001462-67.2025.4.01.3901, of the 732 Indigenous individuals evaluated, 709 (98.5%) exhibited concentrations of one or more toxic or potentially toxic elements exceeding the reference values (****Table 2****). A total of 32 chemical elements were detected, of which 22 metals and semimetals exceeded the reference values adopted in this study, which were defined according to the reference thresholds used by the analytical laboratory and those reported in the public documentation associated with the Civil Public Action. Among these, seven elements are recognized for their toxicological relevance due to absence of an essential physiological role in the human body and their inherent toxicity. These include six metals, Al (detected in 36.5% of individuals), Hg (28.0%), titanium (Ti; 24.0%), Pb (17.0%), barium (Ba; 17.0%), and beryllium (Be) (3.3%), and one semimetal, As (2.8%) [13] (****Figure 1A*** *and* ***B****). Notably, Hg and As are of particular concern, as no safe exposure threshold has been established for either element, and adverse health effects have been reported even at very low levels of exposure. In this study, an element was classified as excessive when its concentration in hair exceeded the reference range established by the BioMinerais Laboratory. Therefore, the values in* ***Table 2*** *refer to the number of elements above reference concentrations for each participant, not the number of elements detected in the sample.*

- Among these, seven elements are considered potentially toxic at high levels of exposure due to their lack of essential physiological function in the human body. These include six metals — Al (found in 36.5% of individuals), Hg (28.0%), titanium (Ti) (24.0%), Pb (17.0%), barium (Ba) (17.0%), beryllium (Be) (3.3%), — and one metalloid, arsenic (As) (2.8%) [11] (Figure 1A and B). - This is INCORRECT, Hg is not POTENTIALLY toxic at high levels, it is ALWAYS toxic at any level, even very low, and As can be toxic at extremely low levels as well.

**Reply:** We thank the reviewer for this important conceptual clarification. We fully agree that mercury has no known safe exposure threshold and is considered toxic even at very low concentrations, and that arsenic can also exert toxic effects at extremely low levels. To address this, we have revised the text to explicitly distinguish elements that are intrinsically toxic (without a recognized safe threshold) from those that become toxic primarily at elevated exposure levels. The description of mercury and arsenic has been corrected accordingly, and an explanatory sentence has been added immediately after the list to clarify that both Hg and As lack a recognized safe exposure limit. This revision improves toxicological accuracy and aligns the terminology with current international consensus.

*In the documentation associated with Civil Public Action n° 1001462-67.2025.4.01.3901, of the 732 Indigenous individuals evaluated, 709 (98.5%) exhibited concentrations of one or more toxic or potentially toxic elements exceeding the reference values (****Table 2****). A total of 32 chemical elements were detected, of which 22 metals and semimetals exceeded the reference values adopted in this study, which were defined according to the reference thresholds used by the analytical laboratory and those reported in the public documentation associated with the Civil Public Action. Among these, seven elements are recognized for their toxicological relevance due to absence of an essential physiological role in the human body and their inherent toxicity. These include six metals, Al (detected in 36.5% of individuals), Hg (28.0%), titanium (Ti; 24.0%), Pb (17.0%), barium (Ba; 17.0%), and beryllium (Be) (3.3%), and one semimetal, As (2.8%) [13] (****Figure 1A*** *and* ***B****). Notably, Hg and As are of particular concern, as no safe exposure threshold has been established for either element, and adverse health effects have been reported even at very low levels of exposure. In this study, an element was classified as excessive when its concentration in hair exceeded the reference range established by the BioMinerais Laboratory. Therefore, the values in* ***Table 2*** *refer to the number of elements above reference concentrations for each participant, not the number of elements detected in the sample.*
